# Screening and Validation of p38 MAPK Involved in Ovarian Development of *Brachymystax lenok*

**DOI:** 10.3389/fvets.2022.752521

**Published:** 2022-02-16

**Authors:** Tianqing Huang, Wei Gu, Enhui Liu, Lanlan Zhang, Fulin Dong, Xianchen He, Wenlong Jiao, Chunyu Li, Bingqian Wang, Gefeng Xu

**Affiliations:** ^1^Key Laboratory of Freshwater Aquatic Biotechnology and Breeding, Ministry of Agriculture and Rural Affairs, Heilongjiang River Fisheries Research Institute, Chinese Academy of Fishery Sciences, Harbin, China; ^2^Heilongjiang Province General Station of Aquatic Technology Promotion, Harbin, China; ^3^Heilongjiang Aquatic Animal Resource Conservation Center, Harbin, China; ^4^Gansu Fisheries Research Institute, Lanzhou, China; ^5^Xinjiang Tianyun Organic Agriculture Co., Yili Group, Hohhot, China

**Keywords:** full-length transcriptome, p38 MAPK, ovarian development, *Brachymystax lenok*, SMRT and NGS

## Abstract

*Brachymystax lenok* (lenok) is a rare cold-water fish native to China that is of high meat quality. Its wild population has declined sharply in recent years, and therefore, exploring the molecular mechanisms underlying the development and reproduction of lenoks for the purposes of artificial breeding and genetic improvement is necessary. The lenok comparative transcriptome was analyzed by combining single molecule, real-time, and next generation sequencing (NGS) technology. Differentially expressed genes (DEGs) were identified in five tissues (head kidney, spleen, liver, muscle, and gonad) between immature [300 days post-hatching (dph)] and mature [three years post-hatching (ph)] lenoks. In total, 234,124 and 229,008 full-length non-chimeric reads were obtained from the immature and mature sequencing data, respectively. After NGS correction, 61,405 and 59,372 non-redundant transcripts were obtained for the expression level and pathway enrichment analyses, respectively. Compared with the mature group, 719 genes with significantly increased expression and 1,727 genes with significantly decreased expression in all five tissues were found in the immature group. Furthermore, DEGs and pathways involved in the endocrine system and gonadal development were identified, and p38 mitogen-activated protein kinases (MAPKs) were identified as potentially regulating gonadal development in lenok. Inhibiting the activity of p38 MAPKs resulted in abnormal levels of gonadotropin-releasing hormone, follicle-stimulating hormone, and estradiol, and affected follicular development. The full-length transcriptome data obtained in this study may provide a valuable reference for the study of gene function, gene expression, and evolutionary relationships in *B. lenok* and may illustrate the basic regulatory mechanism of ovarian development in teleosts.

## Introduction

*Brachymystax lenok* (lenok) is an economically important fish in the Amur Basin with high-quality nutrient-rich meat. As a rare cold water fish native to China, lenok has been listed as a vulnerable species in the Red Book of Endangered Animals in China because of its sharp decline in the wild, and is classified as aquatic wildlife under second-class protection (1). Recent studies on lenok have mainly focused on resource investigation, nutritional physiology, and artificial reproduction (2–4), and studies on the genetic analysis and reproductive regulation mechanisms of lenok are limited because of sparse genetic information. It is important to analyse the molecular mechanisms that regulate lenok reproduction and development to facilitate better breeding and genetic improvement.

The analysis of full-length transcriptome can demonstrate the type and number of genes at the molecular level and is an effective method for revealing the regulatory mechanisms of different physiological and biochemical processes. Many non-model organisms lack reference genome sequence information, and full-length transcriptome sequencing is a rapid and effective method for investigating gene expression, gene function, and evolutionary analysis in this species (5–7). Single molecule real-time sequencing technology (SMRT) based on the Pacific Biosciences (PacBio) platform has the benefits of long reading fragments and high accuracy. Full-length mRNA can be generated directly without assembly, and this technique has been successfully used for the full-length transcriptome analysis of multiple species, such as cattle (8), rabbits (9), mice (10), and shrimp (11). It has also been widely used in teleosts (12–14).

Next generation sequencing based on the Illumina platform is helpful for characterizing various biological processes and exploiting underlying gene activity. Over the past decade, numerous studies have been conducted on genetic and developmental transcriptomics in fish, including microarray and the serial analysis of gene expression. In zebrafish, transcriptome data at nine different stages of embryonic development were comprehensively analyzed to identify the key roles of pathways and functional genes involved in development (15). In haddock (*Atlantic Haddock*), a genetic network for development has been established by analyzing transcriptome data from the embryo to the early developmental stages of juveniles (16). Numerous studies on gonadal development have been conducted using transcriptome sequencing. For example, genes that are differentially expressed during testis development have been characterized in the channel catfish (*Ictalurus punctatus*) (17). In the spotted knifejaw (*Oplegnathus punctatus*) and fugu (*Takifugu rubripe*s), a large number of differential genes were identified in the testes of adult fish compared to the ovaries (18, 19). In gonads from 3–24 weeks after the fertilization (immature to mature) of zebrafish, the dynamic trend of miRNA abundance was characterized by miRNA sequencing (20). However, the number of studies on gonadal development using full length transcriptome methods is limited.

The collection of gonadal tissue samples from lenok could only commence at 300 days post-hatching (dph), and lenok at three years post-hatching (ph) were on the verge of ovulation. These two periods are representative of immature and mature groups for transcriptome sequencing. PacBio and Illumina sequencing were combined to generate two complete full length transcriptomes of immature and mature lenok by analyzing gene expression in five different tissues (liver, muscle, spleen, head kidney, and gonad) and screening for DEGs related to lenok ovarian development. p38 mitogen-activated protein kinases (MAPKs) were defined to express the most significant difference between the immature and mature gonads. MAPKs are a form of conserved serine/threonine protein kinases that participate in the regulation of multiple physiological functions in the form of intracellular signal transmission, and are an important signal transduction system in cells (21). Furthermore, p38 MAPK has been suggested as playing an important role in lenok development and reproduction. As one of the important members of the MAPK family, it is widely expressed in the thyroid, testis, ovary, and pituitary tissues of mammals (22), and plays an important role in the reproductive process (23). However, the role of p38 MAPK in lenok is currently unknown. Therefore, p38 MAPK was selected to demonstrate its role in the balance of reproductive endocrine hormones and the follicular development of lenok. The full-length transcriptome data obtained in this study can provide a valuable reference for further studies on the mechanism of gonadal development and maturation, and make an important contribution to researching the genetic improvement of lenok.

## Materials and Methods

### Ethics Statement

All experiments were performed in accordance with the European Communities Council Directive (86/609/EEC). The experiments were approved by the Animal Husbandry Department of the Heilongjiang Animal Care and Use Committee (202110384464). All fish involved in this research were bred following the guidelines of the Animal Husbandry Department of Heilongjiang, China.

### Fish Sampling and RNA Purification

The gonad (G), head kidney (K), liver (L), muscle (M), and spleen (S) were collected from three mature and 12 immature samples of *B. lenok*, which were bred at the Bohai experimental station of the Heilongjiang River Fisheries Research Institute (129° 04′ 64.7753′′ E; 44° 14′ 5.983′′ N). All samples used in this experiment were obtained from female lenok. The immature group was 300 dph and the mature group was three years ph. Four immature tissue samples were mixed into one RNA sample, which required three repeat RNA samples for sequencing. Therefore, the immature sample size was 12. However, one mature tissue sample can constitute one RNA sample and three duplicate samples require a mature sample size of *N* = 3. Before tissue collection, the fish were euthanized with an overdose of anesthesia in MS-222, as reported previously (2). Each tissue sample was immediately placed into 2 mL sterile tubes and placed in liquid nitrogen. After storing for 1 h, all samples were transferred to a −80 °C refrigerator for further analysis. In addition, gonad samples at 300 dph, 750 dph, and three years ph were used for western blot analysis and were kept at −20 °C. TRIzol reagent (Invitrogen, CA, USA) was used to extract the total RNA, and only RNA samples with a RIN number greater than 7.0 were kept for subsequent experiments. One microgram of each RNA sample was pooled and sequenced using PacBio single-molecule, long-read sequencing (PacBio Sequel, Menlo Park, USA), and Illumina sequencing (Illumina NovaSeq 6000, California, USA) in parallel. The correlation of each sample was R2 > 0.8 (Supplementary Figure S1).

### Complementary DNA (CDNA) Library Construction and PacBio Sequencing

The SMARTer PCR cDNA Synthesis Kit (Takara Bio USA, Inc.) was used to prepare the PacBio cDNA library using the following steps: Mix tube 1 (2 μL total RNA, 1 μL 3'SMART CDS Primer II A, and 1.5 μL deionized H_2_O) at 72 °C for 3 min, and 42 °C for 2 min. Then, mix tube 2 (2 μl 5xFirst-Strand Buffer, 0.25 μl DTT, 1 μl dNTP Mix, 1 μl SMARTer II A Oligonucleotide, 0.25 μl RNase Inhibitor, and 1 μl SMART Scribe Reverse Transcriptase), and then add into tube 1 at 42°C for 1 h, and 72°C for 10 min. The full-length cDNA was subjected to PCR amplification. The quality and concentration of the cDNA library were determined using a Qubit 2.0 Fluorometer and an Agilent 2100 bioanalyzer (24). The 1–6 KB library was sequenced using PacBio Sequel.

### CDNA Library Preparation for Illumina Sequencing

Three replicates for each of the five tissues (gonad, head kidney, liver, muscle and spleen), making a total of 15 RNA samples, were fragmented into small pieces at high temperatures. The mRNA-Seq sample preparation kit (Illumina, San Diego, CA, USA) was used for the reverse transcription of RNA fragments to construct the final cDNA library, and fragments of 250–300 bp were selected using AMPure XP beads (25). The final cDNA library was assessed by PCR and the quality of the cDNA library was determined using an Agilent 2100 Bioanalyzer. Paired-end sequencing was performed on an Illumina NovaSeq 6000, following the recommended protocol (26).

### Data Analysis of PacBio Sequencing

Raw reads were processed into error-corrected reads of inserts (ROIs) using the Iso-seq pipeline (27) with min full pass = 3 and min predicted accuracy = 0.9. Full-length, non-chimeric (FLNC) transcripts were determined by searching for the poly-A tail signal and the 5′ and 3′ cDNA primers in the ROIs (28). Iterative clustering for error correction (ICE) was used to obtain consensus isoforms, and FL consensus sequences from the ICE were polished using Quiver (29). High-quality FL transcripts were classified with a post-correction accuracy above 99%. The cluster database with high identity and tolerance (CD-HIT, http://weizhongli-lab.org/cd-hit/) was used for Iso-Seq high-quality FL transcripts to remove redundancy (identity > 0.99).

### Data Analysis of Illumina Sequencing

Raw reads in Fastq format were first processed using Perl scripts from our laboratory. In this step, clean reads were obtained by removing reads containing adapters, reads containing poly-N, and low-quality reads from the raw data. The Q20, Q30, GC content, and sequence duplication levels of the clean data were concurrently calculated. All downstream analyses were based on high quality clean data. These clean reads were then mapped to the PacBio reference genome sequence. Only reads with a perfect match or one mismatch were further analyzed and annotated based on the reference genome. Hisat2 (30) tools were used to map the reference genome. Gene expression levels were estimated by fragments per kilobase of transcript per million fragments mapped. Prior to differential gene expression analysis, for each sequenced library, the read counts were adjusted using the edgeR (31) program package through one scaling normalized factor. A differential expression analysis of the two samples was performed using the EBSeq R (32) package. The resulting false discovery rate was adjusted using the posterior probability of DE (PPDE). A false discovery rate < 0.01, and fold change ≥ 2 were set as thresholds for significantly differential expression.

### Structure Analysis

Simple sequence repeats (SSRs) in the transcriptome were identified using MISA (http://pgrc.ipk-gatersleben.de/misa/). Candidate coding regions within the transcript sequences were identified using TransDecoder (https://github.com/TransDecoder/TransDecoder/releases). Iso-Seq^TM^ data were directly used to run all-vs-all BLAST (33) with high identity settings. BLAST alignments that met all criteria were considered products of candidate AS events. The coding potential calculator (CPC, http://cpc2.cbi.pku.edu.cn), coding non-coding index (CNCI, https://github.com/www-bioinfo-org/CNCI), coding potential assessment tool (CPAT, http://rna-cpat.sourceforge.net/), and protein family database (Pfam, http://pfam.xfam.org/) were combined to sort non-protein-coding RNA candidates from putative protein-coding RNAs in the transcripts. Transcripts with lengths greater than 200 nt and having more than two exons were selected as long non-coding RNA (lncRNA) candidates.

### Gene Functional Annotation and Enrichment Analysis

Gene function was annotated based on the following databases: NCBI non-redundant protein sequences (NR, http://www.ncbi.nlm.nih.gov/), protein family (Pfam, http://pfam.xfam.org/), Clusters of Orthologous Groups of proteins (KOG/COG/eggNOG, http://www.ncbi.nlm.nih.gov/COG/), Swiss-Prot (a manually annotated and reviewed protein sequence database, http://www.uniprot.org/), Kyoto Encyclopedia of Genes and Genomes (KEGG, http://www.genome.jp/kegg/), and Gene Ontology (GO). The GO enrichment analysis of differentially expressed genes (DEGs) was implemented using the GOseq R (34) packages based on Wallenius non-central hyper-geometric distribution (34), which can adjust for gene length bias in DEGs. KOBAS (35) software was used to test the statistical enrichment of DEGs in KEGG pathways.

### Validation of Expressions of DEGs by Real-Time PCR

Real-time PCR was performed to validate the RNAs involved in RNA-Seq. The same RNA samples were used for the deep sequencing. Twelve mRNAs were detected using real-time PCR. A PrimeScript RT Reagent Kit (TaKaRa, Shiga, Japan) was used for reverse transcription. Real-time PCR was performed using FastStart Universal SYBR® Green Master Mix (Roche, Switzerland) and a CFX96 C1000 touch thermal cycler (Bio-Rad, USA). Beta-actin was used as the reference gene. The Ct values were measured, and the value of the target sequence normalized to the reference sequence was calculated as 2^−ΔΔ^Ct. The statistical analysis was performed using SPSS (36) version 13.0 for Microsoft Windows.

### Western Blot Analysis

Proteins were extracted from gonad samples at 300 dph (immature, *n* = 3) and three years post-hatching (mature, *n* = 3) of female lenoks that were reared under identical conditions to individuals used in transcriptome analyses. To explore the expression profiles of p38 MAPK protein in different tissues, the proteins of the gill, heart, liver, spleen, intestine, skin, and ovary tissues were extracted from female lenok at 750 dph (*n* = 3), which was a time of rapid follicular development. The proteins were boiled for 20 min in SDS-PAGE loading buffer and separated using 12% SDS-PAGE gels. The proteins were transferred to polyvinylidene fluoride membranes (Bio-Rad, USA) and analyzed by western blotting. Anti-p38 MAPK (bs-0637R, Bioss, 1:10000), anti-phospho-p38 MAPK (Thr180) (bs-5476R, Bioss, 1:5000), and beita-actin (ym3121, Immunoway, 1:5000) were used as primary antibodies, and peroxidase-conjugated AffiniPure goat anti-rabbit IgG (1:2000) was used as the secondary antibody (Cell Signaling Technology, USA). After washing with PBST, the protein bands were visualized by infrared fluorescence using the Odyssey Imaging System (LI-COR InC).

### SB203580 Inhibitor Injection

SB203580 (Biorbyt) was used to inhibit the phosphorylation of p38 MAPK. Lenoks of approximately 750 dph were randomly divided into three groups (*n* = 3). CK was the no-treatment group, the negative control (DMSO) was injected intraperitoneally with 1 mL DMSO, and the experimental group (DMSO + I) was injected intraperitoneally with 5 mg/kg SB203580 dissolved in 1 ml DMSO. Seven days post-injection, plasma and gonad samples of the three groups were collected for western blot analysis, hormone level determination, and immunohistochemical assays.

### Reproductive Hormone Level Assay

Polyclonal antibodies were customized based on partial amino acid sequences of follicle-stimulating hormone (FSH), luteinizing hormone (LH), and gonadotropin-releasing hormone 3 (GnRH3) in the lenok. Enzyme-linked immunosorbent assay (ELISA) kits (MLBIO, China) were coated with specific antibodies and developed to detect FSH, LH, and GnRH3 in the lenok. Referring to D'Cotta's method (37), an ELISA was used to verify the specificity and stability of each kit. Ten positive serum samples of each coated antibody were tested, all of which were positive, and 10 blank controls were tested, all of which were negative. The coefficient of variation between and within batches was less than 15%. Standard curves for each ELISA kit are shown in Supplementary Figure S2. The ELISA experiments were performed using a microplate reader at a wavelength of 450 nm. The levels of GnRH3, FSH, LH, and estrogen (E2) were calculated using standard curves. The plasma of three lenoks in CK, DMSO, and DMSO + I groups (*n* = 3) were detected by ELISA, and each sample was assayed three times.

### Immunohistochemistry

Gonad samples from the three groups were fixed in 4% paraformaldehyde for 24 h at 4°C, embedded in paraplast, and sectioned at 5 μm thickness. Paraffin sections were incubated with 3% hydrogen peroxide in phosphate-buffered saline for 10 min, and then blocked with 2% bovine serum albumin and 2% goat serum for 1 h. Sections were boiled with 0.01 M citric acid and EDTA solution for 30 min for antigen recovery. The p-p38 antibody (1:300) was applied to the sections as the primary antibody at 4°C overnight. After washing with phosphate-buffered saline, the sections were incubated with biotinylated goat anti-rabbit secondary antibody (1:1000) for 20 min at 25°C. The sections were then treated with peroxidase-conjugated streptavidin, developed with DAB, and counterstained with haematoxylin. For the negative control, sections were not incubated with the primary antibody. For this part, the gonads of three lenoks from CK, DMSO, and DMSO+I groups (*n* = 3) were analyzed.

### Statistical Analysis

A one-way analysis of variance was used to assess the differences in p38 protein levels in the gills, heart, liver, spleen, intestine, head, skin and gonad, and in gonad at 300 dph, 750 dph, and three years ph. It was also used to assess the differences in reproductive hormone levels in the CK, DMSO, and DMSO+I groups. Data were shown as the mean ± SEM. The statistical analysis was performed using SPSS version 13.0 for Microsoft Windows, and the statistical significance was set at *p* < 0.05.

## Results

### Transcriptome Analysis

#### PacBio Iso-Seq and Bioinformatic Analysis

The PacBio iso-sequencing of lenok was completed, the clean data of mature and immature were obtained by SMRT sequencing technology, and the data sizes were 23.21 GB and 22.67 GB, respectively. Among these, 272,337 and 271,088 circular consensus sequence (CCS) reads were obtained from mature and immature samples, respectively (Table 1), with a mean length of 2,977 and 2,791 bp, respectively, using 37 and 39 passes, respectively, with full passes ≥ 3, and a quality consensus accuracy > 0.9. Subsequently, the CCS reads were classified as full-length non-chimeric (FLNC) with 5′ primer, 3′ primer, and poly-A and non-full length reads, with proportions of 84.09 and 86.36%, respectively, for mature and immature samples. As a result, 229,008 and 234,124 high-quality FLNC reads for mature and immature samples, respectively, were obtained through the cluster of FLNC and correction.

**Table 1:** Statistics of the PacBio Iso-sequencing data.

**cDNA library**	**Mature**	**Immature**
cDNA size	1–6K	1–6K
Date size (GB)	23.21	22.67
CCS number	272,337	271,088
Read bases of CCS	810,860,211	756,750,619
Mean read length of CCS	2,977	2,791
Mean number of passes	37	39
Number of FLNC reads	229,008	234,124
FLNC%	84.09%	86.36%
Number of polished high-quality isoforms	85,270	89,226
Number of polished low-quality isoforms	2,020	2,196
Percent of polished high-quality isoforms	97.25%	97.17%
CD-HIT	61,405	59,372

*GB: A unit used to measure the amount of data. KB: One thousand base pairs*.

We obtained 85,270 and 89,226 high-quality polished consensus sequences (Table 1) for the mature and immature samples, respectively. Finally, 61,405 and 59,372 non-redundant transcripts for mature and immature samples, respectively, were obtained by removing redundant transcripts using CD-HIT. By merging the mature and immature data, we obtained 106,647 non-redundant transcripts for *B. lenok* development analysis. The PacBio Iso-Seq raw data were deposited into the SRA-NCBI repository. The BioProject number was PRJNA669274 and the BioProject number of next-generation sequencing was PRJNA669219.

#### Analysis of Alternative Splicing Events, SSRs and Predictions of Coding Sequences, Transcription Factors, and LncRNAs

**Figure 1:**
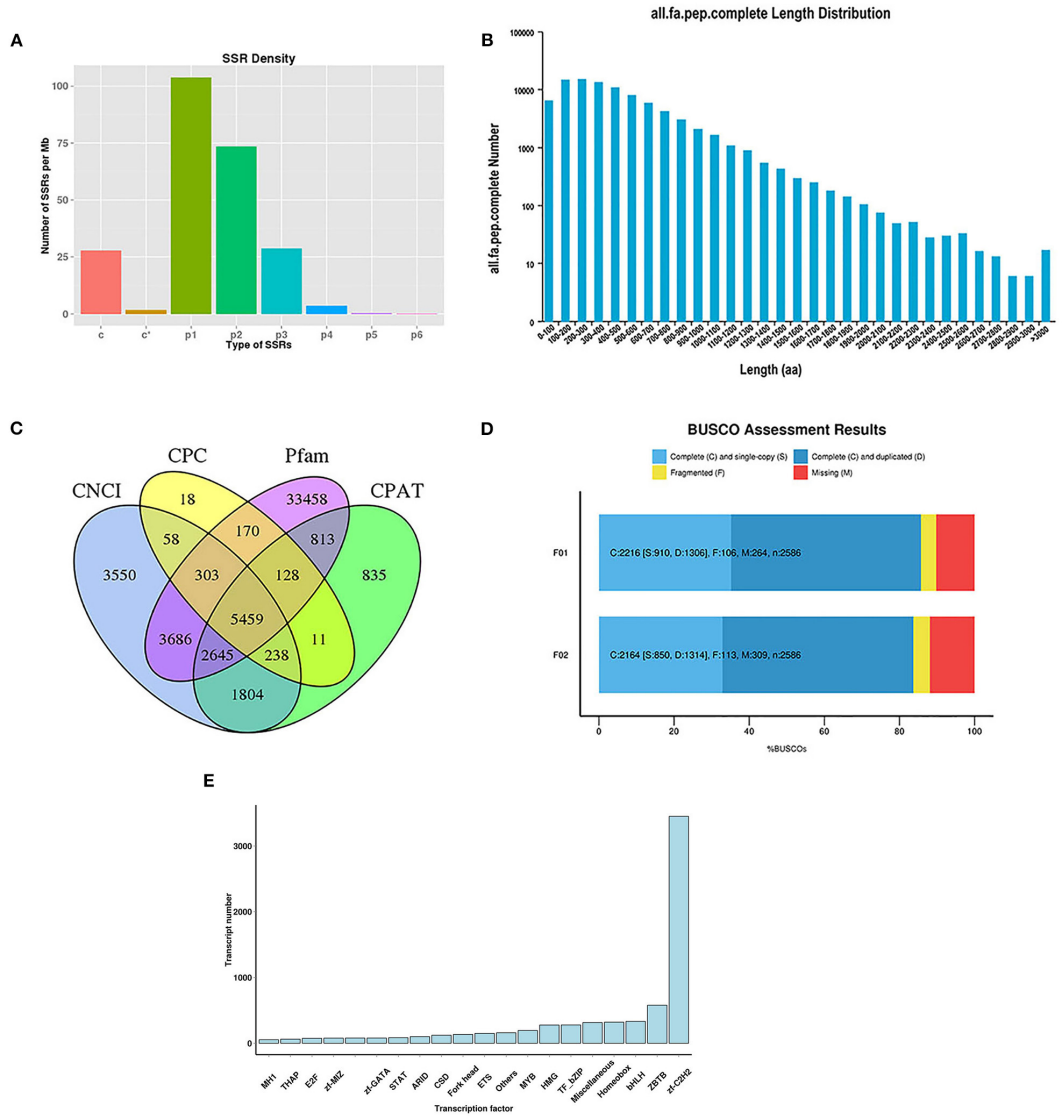
Analysis of SSRs and identification of CDS, TFs, and lncRNAs. **(A)** The density of different type of SSRs; **(B)** The length distribution of predicted CDS; **(C)** Venn diagram for lncRNAs identified by four analysis methods; **(D)** BUSCO assessment results of the completeness of transcripts generated by CD-HIT; **(E)** Distribution of TF families.

In this study, 3,158 and 3,332 alternative splicing (AS) events were analyzed. The absence of a reference genome limited the identification of the AS types. For the analysis of SSRs, 104,373 sequences (344,196,344 bp) were examined, including 103,688 SSRs and 49,478 SSR-containing sequences (Supplementary Table S1). There were 23,300 sequences containing more than one SSR, and the number of SSRs present in the compound form was 21,101. Specifically, most sequences were mononucleotides (42,411), dinucleotides (46,227), and trinucleotides (12,599). The number of different types of SSRs is shown in Figure 1A.

To identify putative protein-coding sequences, we predicted 100,580 open reading frames (ORFs) using the Trans-Decoder. In total, 88,856 coding sequences (CDSs) were identified with start and stop codons. The distributions of the numbers and lengths of complete CDSs are shown in Figure 1B. Among these, 14,579 transcripts (16.41%) were distributed in the 100–200 bp range, 14,893 transcripts (16.76%) in the 200–300 bp range, 13,365 transcripts (15.04%) in the 300–400 bp range, and 10,810 transcripts (12.17%) in the 400–500 bp range.

Here, 5,459 lncRNAs were predicted using a coding potential calculator (CPC), coding-non-coding index (CNCI), Pfam protein structure domain analysis, and coding potential assessment tool (CPAT) (Figure 1C) and candidate lncRNAs for future developmental research on lenok were revealed.

The completeness of the transcripts generated by CD-HIT was assessed using benchmarking universal single-copy orthologs (BUSCO, v.2.3). The results showed that 85.69 and 83.68% of the transcripts of mature and immature lenok samples, respectively, were complete (Figure 1D). Among the mature group, single-copy and duplicated BUSCOs accounted for 41.06 and 58.94%, respectively. In the immature group, the percentages of complete single-copy and duplicated BUSCOs were 39.28 and 60.72%, respectively. Only 106 and 113 fragmented BUSCOs and 264 and 309 missing BUSCOs were found in our two databases (Figure 1D). These results all show that our database is complete and available for subsequent research.

In total, 7,628 putative transcription factors (TFs) were examined by sequencing, and the top 20 families with the highest number of TFs are shown in Figure 1E. Most TFs belonged to the zf-C2H2 (3,450), ZBTB (579), bHLH (333), homeobox (361), miscellaneous (315), TF-bZIP (279), and HMG (278) families.

#### Function Annotation of Transcripts

The annotation information of 102,295 (95.92%) non-redundant transcripts was obtained by blasting eight databases, namely, NR, Swiss-Prot, COG, KOG, Pfam, eggNOG, GO, and KEGG. The number of annotated transcripts in each database is listed in Table 2.

**Table 2:** Number of annotated transcripts of each database.

**Annotated databases**	**Number of transcripts**
COG	33,077
GO	83,729
KEGG	67,965
KOG	74,798
Pfam	88,168
Swiss-Prot	69,821
eggNOG	99,079
NR	102,107
All	102,295

**Figure 2:**
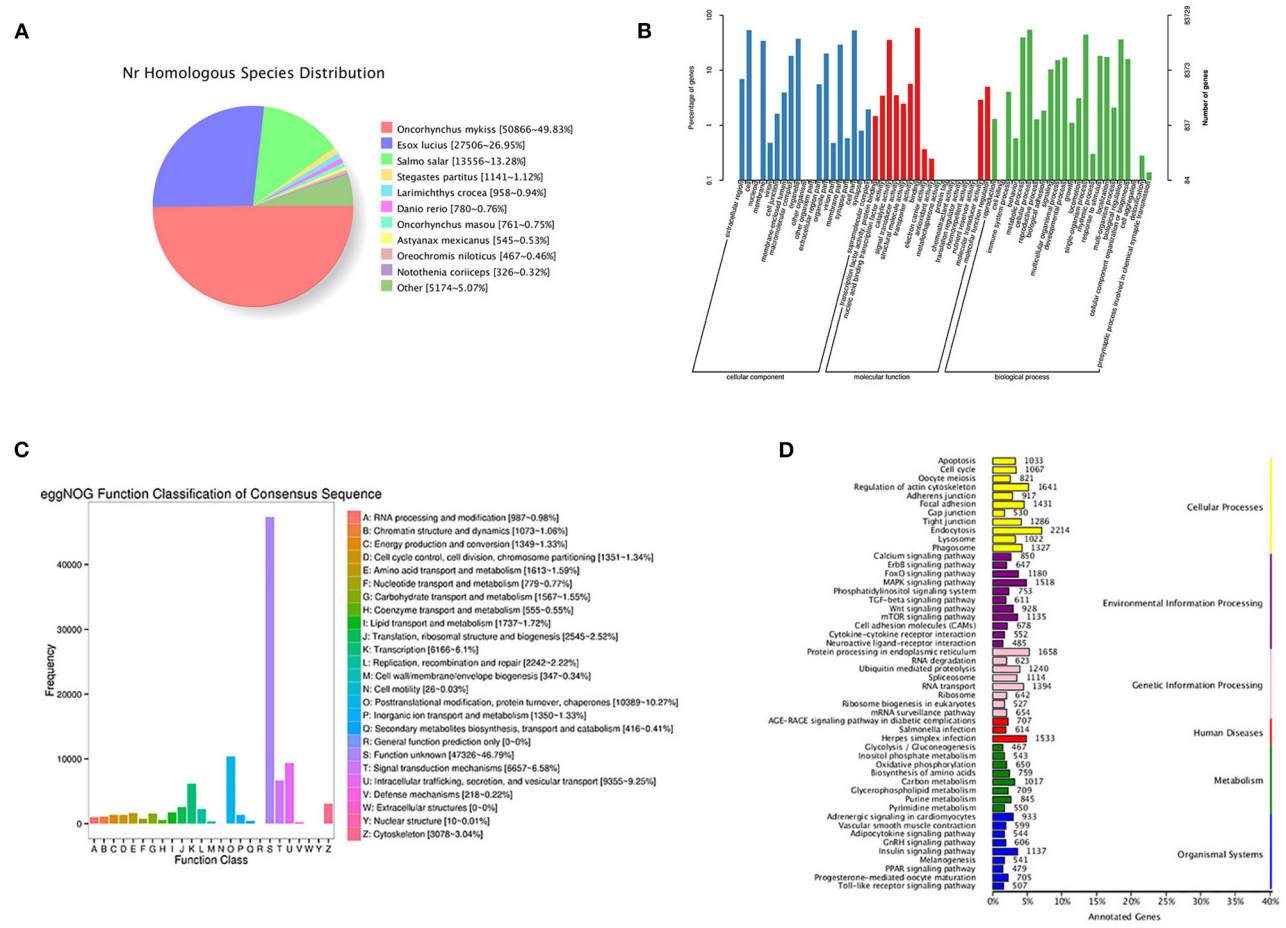
Functional annotation information of non-redundant transcripts. **(A)** Homologous species distribution of transcripts in NR database; **(B)** GO analysis of annotated transcripts; **(C)** eggNOG function classification of transcripts; **(D)** KEGG pathway classified analysis of transcripts.

By blasting the sequences of homologous species in the NR database, 50.866 (49.83%) annotated transcripts were aligned with *Oncorhynchus mykiss*, followed by *Esox luclus* (26.95%) and *Salmo salar* (13.28%) (Figure 2A).

In total, 83,729 transcripts were enriched in the three ontologies based on the GO analysis (Figure 2B). In the cellular component (CC), transcripts were mainly enriched in cells (44,531), cell parts (44,364), and organelles (31,422). Most transcripts enriched in molecular processes (MF) were binding (49,208), catalytic activity (29,970), and transporter activity (4,766). For biological processes (BP), transcripts were mainly enriched in cellular processes (45,934), single-organism processes (37,336), and metabolic processes (33,199).

In this study, 99,079 transcripts were annotated by blasting with linear homologous groups in the eggNOG database for function description and classification. The annotation results were classified into 25 categories (Figure 2C). The three largest groups were post-translational modification, protein turnover, chaperones (10,389), intracellular trafficking, secretion, vesicular transport (9,355), and signal transduction mechanisms (6,657) followed by transcription (6,166), cytoskeleton (3,078), and translation, ribosomal structure, and biogenesis (2,545).

Based on the KEGG analysis, 67,965 transcripts were enriched in 297 pathways. The five pathways enriched with the most genes were *endocytosis* (2,214), *protein processing in endoplasmic reticulum* (1,658), *regulation of actin cytoskeleton* (1,641), *herpes simplex infection* (1,533), and *MAPK signaling pathway* (1,518) (Figure 2D).

#### Differentially Expressed Genes (DEGs) Analysis

The expression levels of genes in five tissues of immature and mature lenok were investigated, including the gonad (G), head kidney (K), liver (L), muscle (M), and spleen (S). The numbers of upregulated and downregulated DEGs between the immature and mature groups are shown in Table 3.

**Table 3:** Statistics for DEGs of five tissues between of immature and mature *Brachymystax lenok*.

**DEG Set (immature vs. mature)**	**All DEGs**	**Up-regulated**	**Down-regulated**
Gonad vs. M gonad	7,728	3,306	4,422
Head kidney vs. M head kidney	10,352	3,840	6,512
Liver vs. M liver	6,690	2,254	4,436
Muscle vs. M muscle	7,968	3,268	4,700
Spleen vs. M spleen	10,837	4,089	6,748

**Figure 3:**
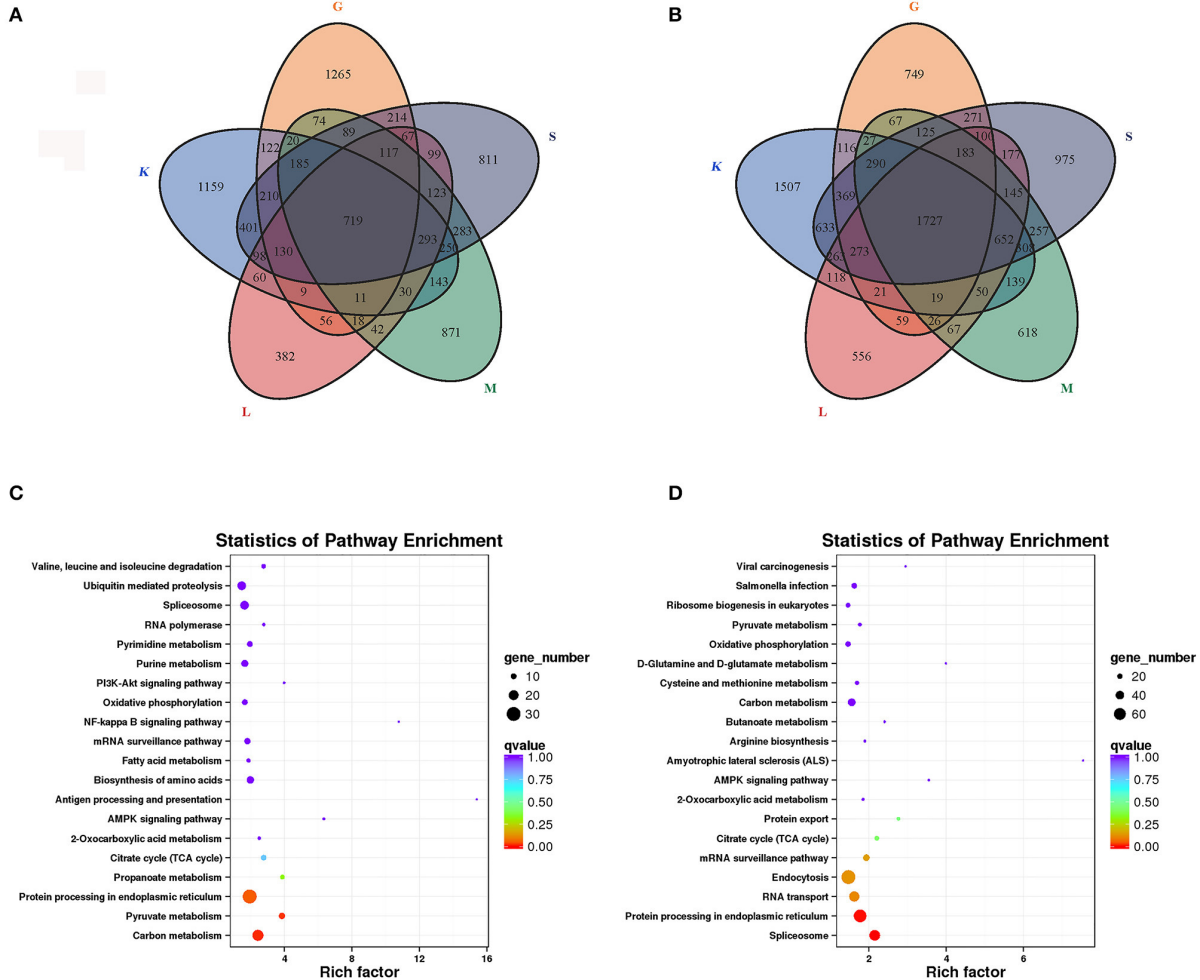
Expression analysis of DEGs in five tissues of lenok. **(A)** Upregulated DEGs; **(B)** Downregulated DEGs; K, head kidney; L, liver; M, muscle; S, spleen; G, gonad; **(C)** KEGG analysis of upregulated DEGs; **(D)** KEGG analysis of downregulated DEGs.

We compared the DEGs between the immature and mature groups in the five tissues. Compared with the mature group, 719 genes with significantly increased expression (Figure 3A) and 1,727 genes with significantly decreased expression (Figure 3B) in all five tissues were found in the immature group. To investigate the function of DEGs, a KEGG analysis was performed on the overlapping genes. The results showed that 291 of 719 upregulated overlapping DEGs were enriched in the KEGG pathway, among which the significantly enriched pathways were carbon metabolism (23), pyruvate metabolism (11), and protein processing in the endoplasmic reticulum (30) (Figure 3C). Among the 1,727 downregulated overlapping DEGs, 693 genes were enriched in the KEGG pathway, among which the enriched pathways included spliceosome (53) and protein processing in the endoplasmic reticulum (65) (Figure 3D).

#### Pathways Related to Endocrine System and Development

**Figure 4:**
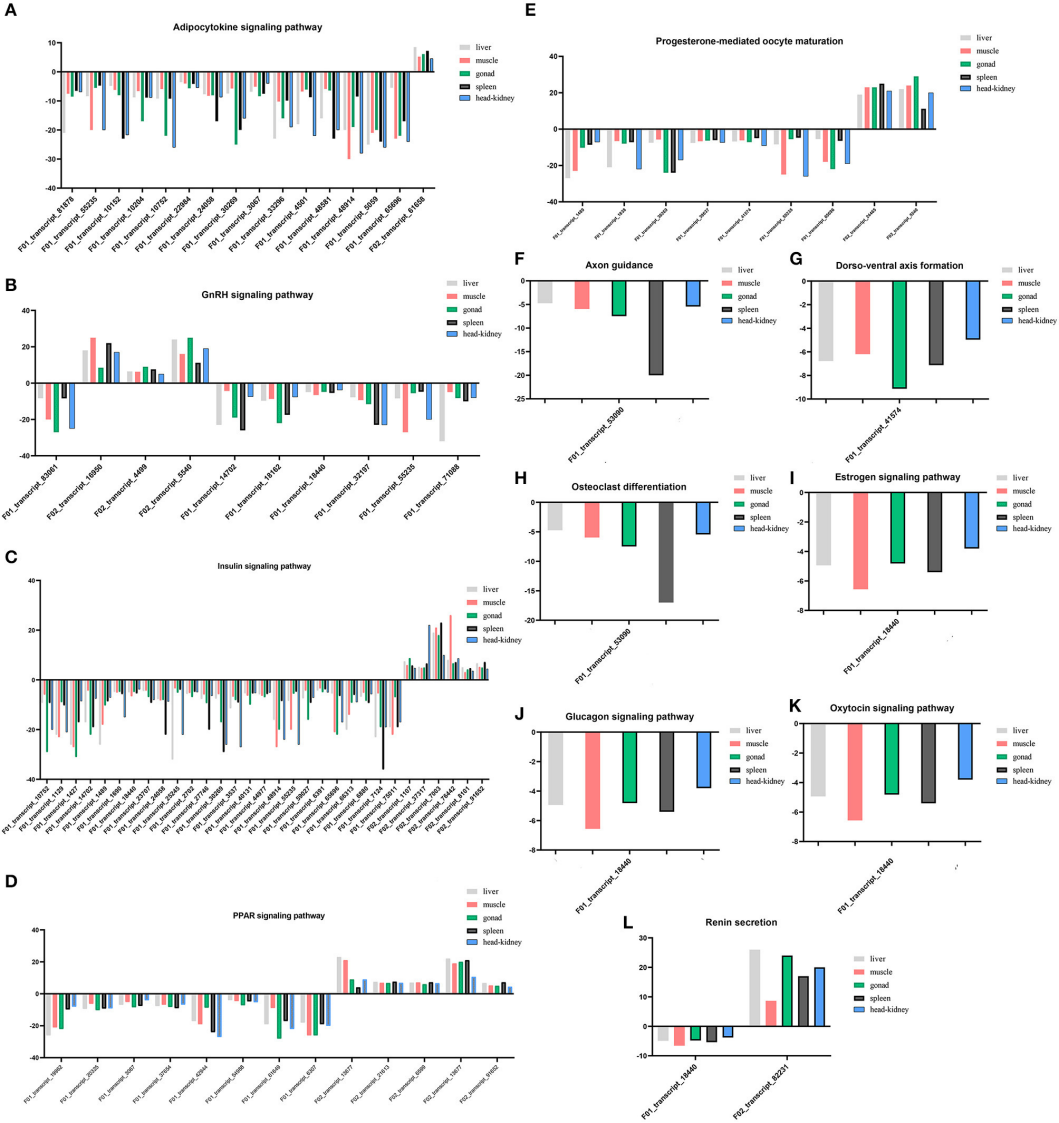
**(A–L)** Expression profiles of DEGs enriched in pathways involved in endocrine system and development in five tissues.

The endocrine system and development-related pathways play important roles in the process of gonadal maturation (38). According to the classification information of the KEGG database (https://www.kegg.jp/kegg/pathway.html), the second classifications of endocrine system and development were under the primary classification of organismal systems. We analyzed the enriched DEGs in the pathways related to the endocrine system and development and their expression patterns in different tissues of immature and mature groups. A total of 77 DEGs were screened for the enrichment of these two classifications, and they enriched the *adipocytokine signaling pathway* (ko04920), *GnRH signaling pathway* (ko04912), *insulin signaling pathway* (ko04910), *PPAR signaling pathway* (ko03320), *progesterone-mediated oocyte maturation* (ko04914), and *renin secretion* (ko04924). Information regarding the DEGs enriched in these pathways is presented in Supplementary Table S2, and their expression profiles in different tissues are shown in Figure 4. There were 31 DEGs enriched in the *insulin signaling pathway*, followed by the *adipocytokine signaling pathway* [16], *PPAR signaling pathway* [12], *GnRH signaling pathway* [10], *progesterone-mediated oocyte maturation* [9], *renin secretion* [2], and only one DEG was enriched in the remaining pathways.

Among the 77 DEGs enriched in the above pathways, the expression of 69 DEGs increased in mature lenok, indicating that the expression of genes involved in development and the endocrine system increased with the growth and development of lenok. Among the DEGs enriched in the above pathways, the five genes that expressed the most significant differences in the gonads were F01_transcript_1427 (unknown function), F02_transcript_5540 (p38 mitogen-activated protein kinase), F01_transcript_61649 (unknown function), F01_transcript_83061 (guanine nucleotide-binding protein subunit alpha-11), and F01_transcript_10752 (5-AMP-activated protein kinase) (Table 4).

**Table 4:** Specifically expressed genes of the top five |log2FC| in gonad.

**Gene**	**|log2FC|**	**Description**
F01_transcript_1427	−31.50	Protein of unknown function
F02_transcript_5540	−29.78	p38 mitogen activated protein kinase
F01_transcript_61649	−28.96	Protein of unknown function
F01_transcript_83061	−26.47	Guanine nucleotide-binding protein subunit alpha-11
F01_transcript_10752	29.37	5'-AMP-activated protein kinase

#### Verification of RNA-seq by qRT-PCR

**Figure 5:**
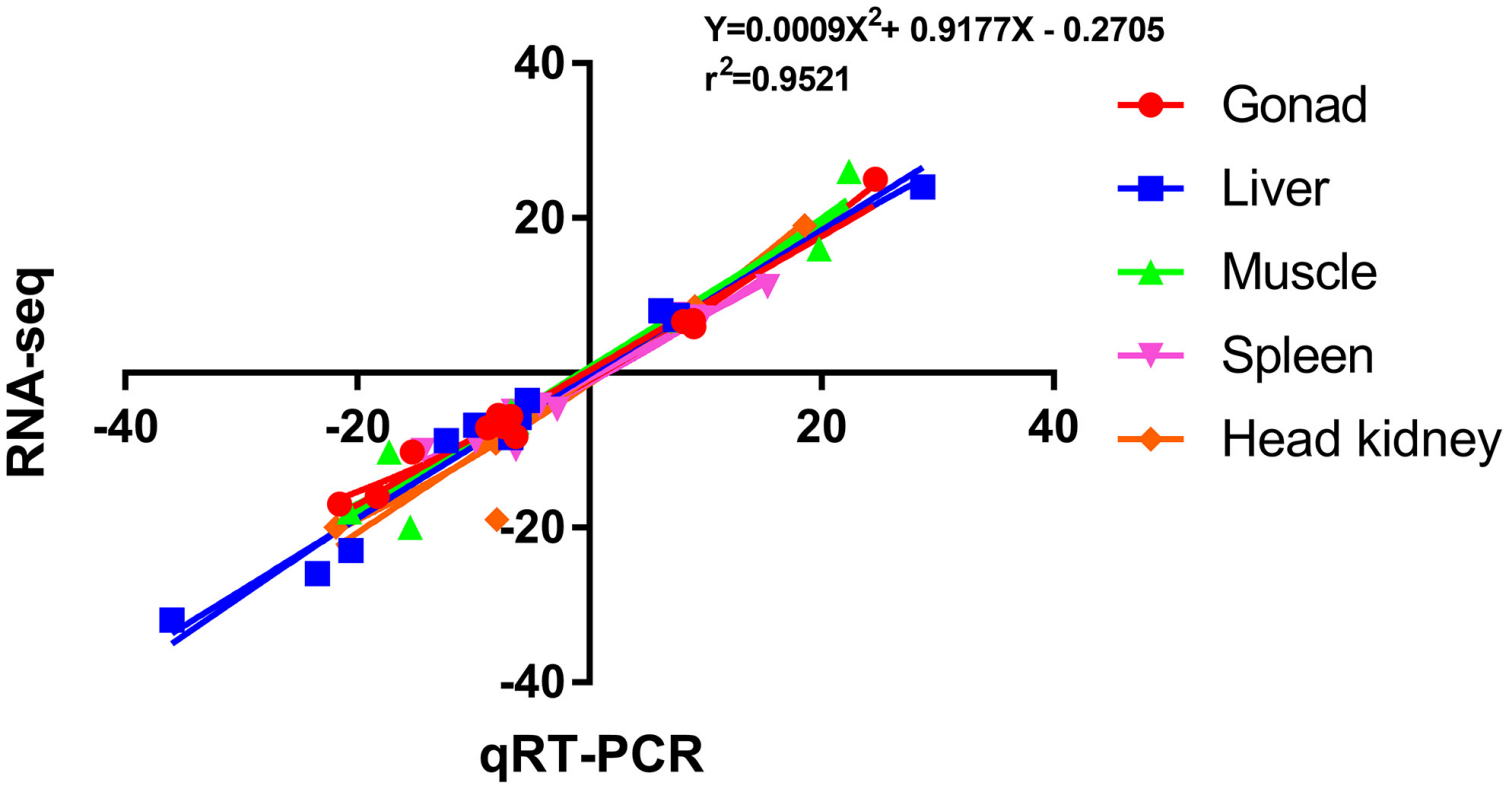
Scatterplot of the correlation between qRT-PCR and RNA-seq. X-axis, qRT-PCR; Y-axis, RNA-seq; r2, Pearson correlation coefficient.

To verify the accuracy of RNA-seq, 12 DEGs were randomly selected for qRT-PCR verification, and their primer information is presented in Supplementary Table S3. The results of RNA-seq and qRT-PCR of these 12 DEGs in the five tissues are shown in Figure 5, and the correlation was expressed by Pearson's coefficient (r^2^ = 0.9521). The results showed consistency and correlation between the results of RNA-seq and qRT-PCR, which proved the effectiveness of RNA-seq.

### Validation Analysis

#### Expression Analysis of p38 MAPK in *B. lenok*

**Figure 6:**
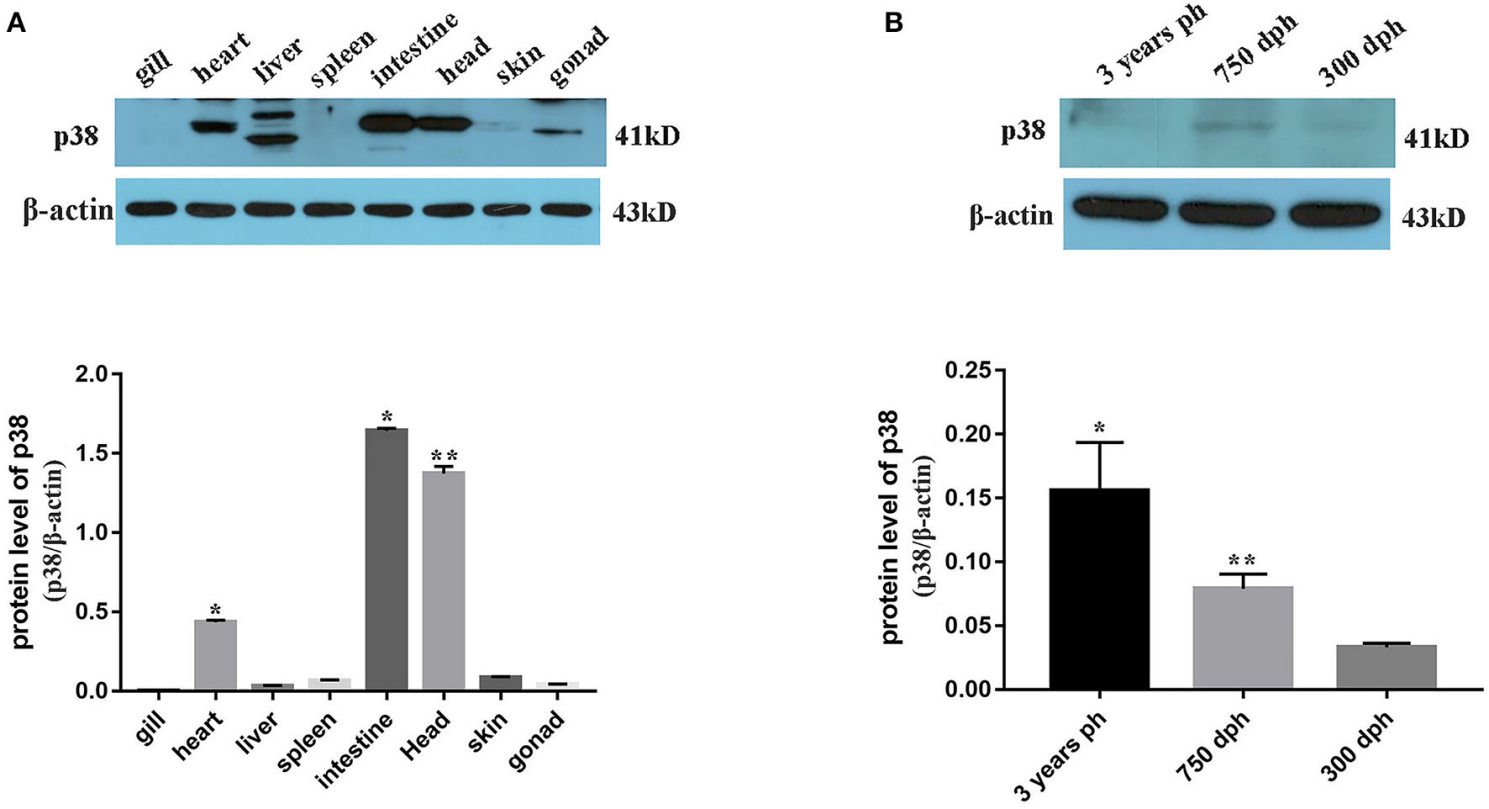
Expression pattern of p38 MAPK protein in *B. lenok*. **(A)** The expression level of p38 MAPK protein in 8 tissues. The significance was expressed “*” as a *P*-value of < 0.05 vs. gill, and “**” as a *P*-value of < 0.01 vs. gill. **(B)** The expression level of p38 MAPK protein in gonad at different developmental stages. The significance was expressed “*” as a *P*-value of < 0.05 vs. 300 dph, and “**” as a *P*-value of < 0.01 vs. 300 dph.

Transcriptome sequencing results showed that p38 MAPK was significantly differentially expressed in mature and immature gonads (Table 4). To study the expression pattern of p38 MAPK in lenok, the expression of p38 MAPK protein was detected in eight tissues of lenok, including the gill, heart, liver, spleen, intestine, brain, skin, and gonads (Figure 6A), and the results showed that the expression of p38 MAPK was higher in the intestine, heart, and brain tissues, but was not expressed in the gill and spleen. The expression levels of p38 MAPK protein were also detected in the ovaries of lenok at different developmental stages. The results showed that the expression of p38 MAPK at 750 dph was 2.8-fold that at 300 dph, and the expression at three years ph was 2.0-fold of that at 750 dph and 3.1-fold of that at 300 dph (Figure 6B). The results showed that the expression of p38 MAPK in the ovary significantly increased with the growth and development of lenok.

#### Effects of p38 MAPK on Ovarian Development of *B. lenok*

**Figure 7:**
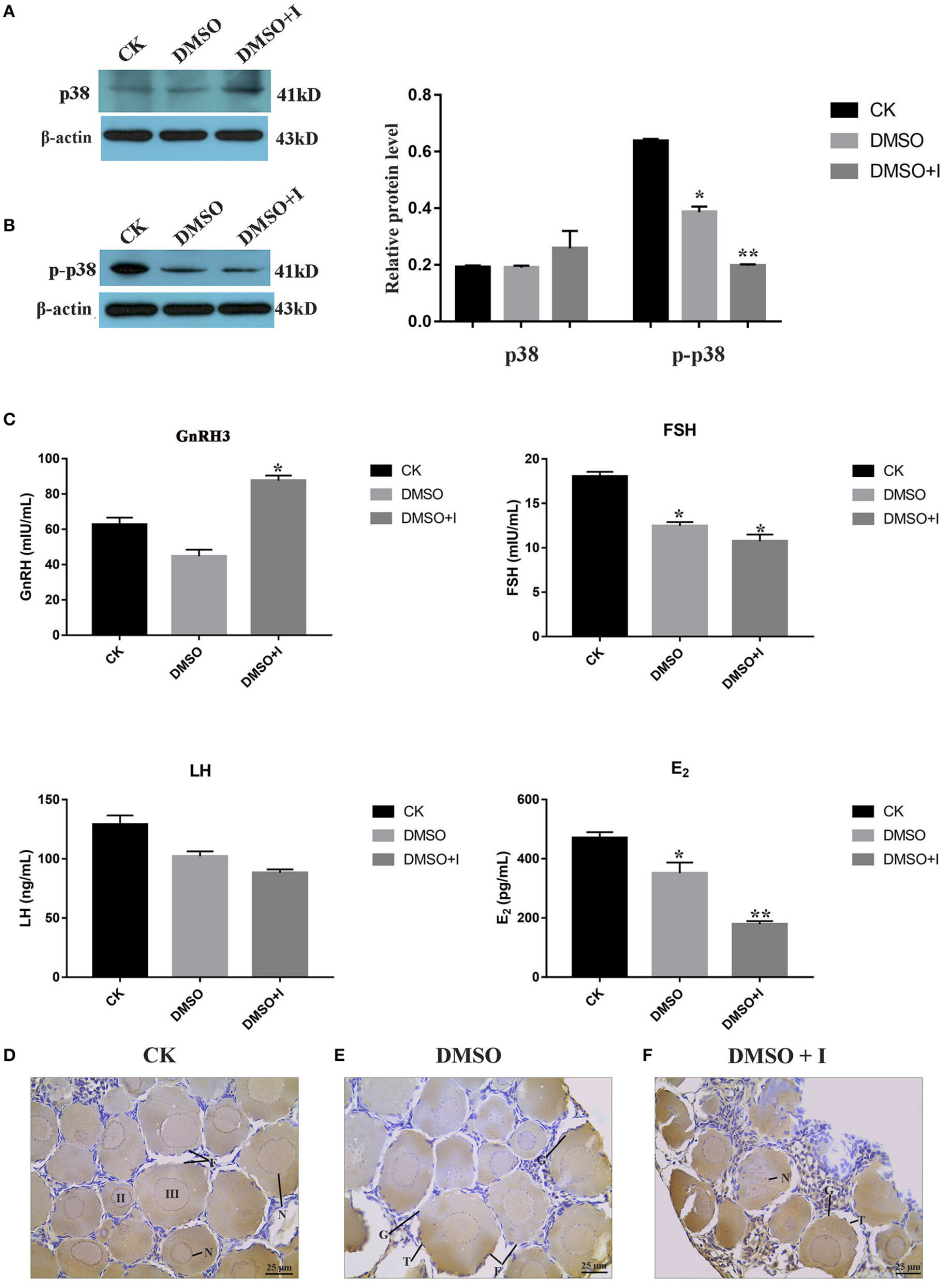
Effects of p38 MAPK on ovarian development of *B. lenok*. **(A)** The expression level of p38 MAPK protein in ovary; **(B)** The phosphorylation level of p38 MAPK protein in ovary of different groups; **(C)** The level of HPG hormones of different groups; The significance was expressed “*” as a *P*-value of < 0.05 vs. CK group, and “**” as a *P*-value of < 0.01 vs. CK group. **(D–F)** Ovarian morphology of different groups observed by immunohistochemistry. II, oocyte of phaseII; III, oocyte of phase III; F, follicle; T, theca cell; G, granulosa cell; N, nucleus.

To verify the regulatory effect of p38 MAPK on ovarian development in lenok, an inhibitor of SB203580 was injected intraperitoneally to inhibit p38 MAPK protein phosphorylation. The results showed that the expression levels of p38 MAPK protein in the SB203580 inhibitor, DMSO, and control groups were not significantly different (Figure 7A). However, the phosphorylation levels of p38 in the SB203580 inhibitor group were 0.32-fold that of the control group and 0.55 times that of the DMSO group, indicating a significant decreasing trend (Figure 7B).

To explore the effect of the p38 MAPK pathway on the balance of hormones in the hypothalamic-pituitary-gonadal (HPG) axis of lenok, gonadotropin-releasing hormone 3 (GnRH3), follicle stimulating hormone (FSH), luteinizing hormone (LH), and oestradiol (E2) hormone levels were detected in the plasma of lenok in the SB203580 inhibitor, DMSO, and control groups, respectively. The values of OD450 in different groups are shown in Supplementary Table S4. The repetition rate of the GnRH3 assay was 1.7–7.8%, the FSH was 1.9–9.6%, the LH was 1.3–4.7%, and the E2 was 1.8–7.8%. The variable coefficients of all samples were no greater than 10%, proving that our ELISA results had good repeatability.

The results showed that the level of GnRH3 in the inhibitor group was 1.4-fold higher than that in the control group (*P* < 0.05) and 1.9-fold higher than that in the DMSO group (*P* < 0.05), and there was no significant difference between the control and DMSO groups (*P* > 0.05). The FSH level in the inhibitor group was 0.6-fold lower than that in the control group (*P* < 0.05), and there was no significant difference between the inhibitor and DMSO groups (*P* > 0.05). The LH levels in the three groups were not significantly different (*P* > 0.05). The level of E2 in the inhibitor group was significantly lower than that in the control and DMSO groups, which was 0.4-fold higher than that in the control group (*P* < 0.01) and 0.5-fold higher than that in the DMSO group (*P* < 0.05) (Figure 7C).

Immunohistochemical sections labeled with p38 MAPK protein were used to explore the morphological changes in lenok ovaries after p38 MAPK activity was inhibited. There were oocytes at phases II and III in the control group. The cell volume was larger, and the cytoplasm increased with the nucleus inside. A flat layer of nucleolar structure overlapped the oocyte and the theca cells around the follicle were complete (Figure 7D). In the DMSO group, the morphology of follicles was almost consistent with that of the control group, and only theca cells appeared irregular (Figure 7E), which may be because of the toxic effect of DMSO on the ovary. In the inhibitor group, the structure of follicles was obviously changed, with the rupture of cell nucleus, vacuoles in follicles, and atrophy of theca cells, which showed irregular arrangement (Figure 7F).

## Discussion

In this study, SMRT technology based on the PacBio platform was used to sequence the mixed tissue samples of immature and mature lenok, including the liver, muscle, head kidney, gonad, and spleen. SMRT technology is superior to next-generation sequencing technologies such as Illumina, which can conduct de novo sequencing of the complete mRNA to obtain the full-length information of the transcripts directly. This method can provide accurate reference sequences, overcome the problems of short transcription splicing and incomplete information of species without reference genomes, and contribute to the screening and identification of functional genes. As a rare cold-water fish native to China, the wild population of *B. lenok* is decreasing annually. The analysis of genetic mechanisms plays an important role in the establishment of artificial breeding technology and genetic improvement of lenok. As a non-model organism, the identification of functional genes in lenok is limited because of the lack of a reference genome. In this study, key genes and pathways related to the gonadal development of lenok were screened using SMRT sequencing and corrected using Illumina sequencing, and the function of the gene involved in ovarian development of lenok was explored.

The Iso-seq results showed that the lengths of the circular consensus sequence (CCS) reads were 2,791 and 2,977, respectively, and the percentage of FLNC reads with the 5′ end, 3′ end, and poly-A structures were 84.09 and 86.36%, respectively, for mature and immature samples. The length of N50 and proportion of FLNC were better than those of red swamp crayfish (39) and Hong Kong catfish (40). After NGS correction, 61,405 and 59,372 non-redundant transcripts were obtained for the mature and immature groups, respectively, for subsequent expression levels and pathway enrichment analysis. These transcripts were evaluated by BUSCO, and the transcripts that encoded complete proteins accounted for 85.69 and 83.68% in the mature and immature groups, respectively. The proportion of complete transcripts in this study was higher than that obtained from the full-length transcriptome of other aquatic animals, such as Atlantic bluefin tuna (80%) (41) and shrimp (81%) (11). These results proved that our sequencing results were of high quality and were reliable for the analysis of functional gene information.

Eukaryotic transcription factors can specifically bind to the upstream sequence of the 5'-end of a specific gene, thus ensuring that the gene is expressed at a specific intensity at a specific time and space (42). Studies have shown that TFs play important roles in fish morphogenesis (43), growth and development (44), gonadal maturation (45), and immune regulation (46). The sequencing results of the full-length transcriptome in this study showed that the TFs of the zf-C2H2, ZBTB, BHLH, homeobox, miscellaneous, TF-BZIP, and HMG families appeared in large numbers, suggesting that they play pivotal roles in the growth and development of lenok. The C2H2 zinc finger (zf-C2H2) proteins are the most abundant transcriptional regulatory factors in mammals. Most of the zinc finger motifs of zf-C2H2 proteins are not conserved, indicating that they may bind to different DNA sequences to regulate different genes and perform diverse regulatory functions. Most human zf-C2H2 proteins are completely different from those of other species, such as mice, so the function of zf-C2H2 proteins is not consistent between different species (47). In this study, we concluded that the zf-C2H2 protein was the most abundant type of TF in lenok, and its specific function should be further studied. The ZBTB family refers to a class of proteins containing the N-terminal BTB domain and multiple zinc finger domains at the C-terminal (48), and more than 60 types of ZBTB proteins have been identified as being involved in development, differentiation, and tumor formation (49). Recent studies have shown that *zbtb16* can regulate spermatogenesis by controlling the self-renewal and repair of spermatogonia (50, 51). In orange-spotted grouper, *zbtb40* is specifically expressed in male germ cells and regulates spermatogenesis through its interaction with *cyp17a1* (49).

Different tissues play important regulatory roles in the growth and development of fish. Through transcriptome screening, DEGs were identified in the gonad, head kidney, liver, muscle, and spleen tissues between mature and immature lenok groups. Most DEGs were found in the spleen (10,837), followed by the head kidney (10, 352), muscle (7,968), gonad (7,728), and liver (6,690). The spleen and head kidney are important haematopoietic tissues in fish and play important roles in the generation, storage, and maturation of red blood cells and granulocytes (52, 53). The head kidney also contains phagocytes and B cells, and is an important organ for the production of antibodies (54). In many fish, the ability of specific immunity gradually increases with growth and development (55), and therefore, the number of DEGs in the spleen and head kidney was higher. Compared with the mature group, the number of downregulated DEGs was higher than that of the upregulated DEGs in the immature group, which indicated that the expression of most genes showed a significantly increased trend with the growth and development of lenok.

Due to the rapid decline in natural resources, it is of great practical significance to conduct the artificial reproduction and genetic breeding of lenok. Therefore, it is of great importance to understand the regulatory mechanisms of gonadal development and maturation of lenok. Gonadal developmental processes, such as germline generation, proliferation, and yolk accumulation in fish are regulated by the endocrine system (56). DEGs enriched in pathways involved in the endocrine system were analyzed, and most DEGs were found to be enriched in the *insulin signaling pathway* (ko04910) and the *peroxisome proliferator-activated receptor (PPAR) signaling pathway* (ko03320). Insulin plays an important role in the reproductive process of female animals and has direct and indirect effects on the production of ovarian steroid hormones and the growth of granulosa and theca cells (57). There are many important effectors in the insulin pathway, including insulin receptor (IR), insulin receptor substrate (IRS), phosphatidylinositol 3 kinase (PI3K), and protein kinase B (AKT). IR expression has been detected in granulosa and theca cells and follicles in a variety of animals (58–60) at different stages of development (61). PI3K can regulate Akt to participate in many physiological processes, and the PI3K/Akt pathway plays key regulatory roles in follicular structural differentiation, growth, and development (62). In this study, we preliminarily showed that the insulin signaling pathway plays an essential role in the regulation of the ovarian development in lenok. PPARs are important in the reproductive system, particularly in the HPG axis (63). Some studies have demonstrated that PPARs can regulate ovum proliferation, tissue remodeling, and hormone synthesis (64).

The expression patterns of DEGs enriched in endocrine and development-related pathways were analyzed in the gonads of lenok. The results indicated that p38 MAPK, GNA11, and AMPK were significantly differentially expressed between mature and immature gonads, but the functions of these genes in lenok remain unknown. As a central substance of cell energy metabolism, adenylate activated protein kinase (AMPK) plays a non-negligible role in the process of ovarian development (65). Glucose and adiponectin promote AMPK phosphorylation (66), whereas IGF-1 and FSH inhibit AMPK phosphorylation (67). Phosphorylated AMPK promotes oocyte development, but negatively regulates follicle and granulosa cell development (68). As one of the main branches of the MAPK signaling pathway, p38 MAPK can be activated in response to a variety of environmental stresses or inflammatory stimuli, thereby promoting apoptosis and inhibiting cell growth (22). However, research on lenok remains in its infancy. In this study, the expression of p38 MAPK protein was detected in different tissues of lenok, and the results showed that p38 MAPK plays a role in the regulation of intestinal, heart, and brain tissues. The expression pattern of p38 MAPK protein was simultaneously explored at different developmental stages of the lenok ovary. Under the same artificial feeding conditions, the expression levels of p38 MAPK protein increased gradually with the growth and development of lenok.

SB203580 is a pyridine imidazole derivative that can inhibit the catalytic activity of p38 MAPK by binding competitively to the ATP sites (69), and thus, can specifically inhibit the p38 MAPK signaling pathway (70, 71). The inhibitor was injected intraperitoneally to investigate the effects of p38 MAPK on steroid hormone levels and follicular structures of lenok. The results showed that under the influence of the inhibitor SB203580, the phosphorylation levels of p38 MAPK in the ovary were significantly decreased, indicating that SB203580 blocked the p38 MAPK signaling pathway. We also investigated the influence of p38 MAPK pathway inhibition on the HPG axis of lenok. The synthesis and secretion of GnRH in the hypothalamus promotes LH and FSH synthesis and secretion in the pituitary, thereby stimulating the synthesis and secretion of E2 in the ovary (72). Hormones in the HPG axis maintain normal development of the ovary and oogenesis and regulate the physiological functions of the reproductive system of fish (73). The levels of major reproductive hormones in the plasma were monitored to explore the effects of p38 MAPK signaling pathway inhibition on lenok reproductive hormone balance. Oocytes at 300 dph (immature) ovaries were in the early stage, while a large amount of yolk existed in follicles at three years ph (mature), which would have a substantial influence on histological experiments. However, oocytes of various stages existed in the ovary of lenok at 750 dph, which was more suitable for function verification.

GnRH is synthesized and secreted by GnRH neurons in the medial hypothalamus and maintains hormone balance and homeostasis through autocrine and paracrine mechanisms under the synergistic effect of related hormones (74–76). Compared with the CK and DMSO groups, the GnRH3 level in the inhibitor group increased significantly, indicating that the hormone level of the HPG axis was abnormal, resulting in the continuous secretion of GnRH3 hormone in the hypothalamus. However, no serious tissue damage was caused to the hypothalamus. FSH and LH are secreted by the anterior pituitary gland, and FSH binds to specific receptors and stimulates the formation of LH receptors (77, 78). In addition, FSH acts synergistically with LH to promote follicle maturation and stimulate E2 synthesis and secretion in the ovary (79, 80). In this study, it was found that compared with the CK group, FSH levels were significantly decreased in the DMSO group, which may be caused by toxicity of DMSO. In the inhibitor group, FSH levels were significantly decreased, whereas there was no significant effect on LH levels. Previous studies have shown that inhibition of the p38 MAPK pathway can weaken the stimulatory effect of FSH on E2 and cytochrome P450 aromatase (81). Combined with the results of this study, it was confirmed that the p38 MAPK pathway is involved in the synthesis and function of FSH in lenok.

E2 is a steroid hormone produced by follicular theca cells and is the main estrogen of teleosts (82). It plays important reproductive roles, such as promoting follicular development and regulating the oestrus cycle (83). The results of this experiment showed that the level of E2 in the inhibitor group was significantly lower than that in the CK group. Previous studies have shown that inhibition of p38 MAPK activity can inhibit the generation of E2 (84). In addition, combined with the results of immunohistochemistry in this study, inhibition of p38 MAPK activity could cause atrophy and irregular arrangement of granulosa and theca cells, where E2 was mainly secreted. Thus, inhibition of p38 MAPK activity could result in morphological and functional changes in the granulosa and theca cells of lenok and affect the synthesis and secretion of E2, leading to HPG axis blocking and abnormal follicular development. Therefore, the p38 MAPK pathway plays an important role in maintaining the balance between reproductive hormones and follicle development in lenok.

## Conclusion

This is the first comparative transcriptome analysis of *B. lenok* combined with SMRT and NGS, and for the first time, the DEGs between immature and mature lenok were analyzed in five tissues. Furthermore, DEGs and pathways involved in the endocrine system and gonadal development were identified, and p38 MAPK was identified to potentially regulate gonadal development in lenok. Inhibiting the activity of p38 MAPK blocked the HPG axis and abnormal follicular development in lenok. Our study illustrates the basic regulatory mechanism of ovarian development and provides a reference for genetic improvement in lenok.

## Data Availability Statement

The datasets presented in this study can be found in online repositories. The names of the repository/repositories and accession number(s) can be found below: https://www.ncbi.nlm.nih.gov/, PRJNA669274 and https://www.ncbi.nlm.nih.gov/, PRJNA669219.

## Ethics Statement

The animal study was reviewed and approved by all experiments were performed according to the European Communities Council Directive (86/609/EEC). The performances were approved by the Animal Husbandry Department of Heilongjiang Animal Care and Use Committee (202110384464).

## Author Contributions

TH designed and performed the experiments. GX analyzed the data and checked the manuscript. WG, LZ, WJ, and CL cultured and sampled the fish. EL and FD drafted the manuscript. XH and BW reviewed the manuscript. All authors contributed to the article and approved the submitted version.

## Funding

This study was supported by the China Agriculture Research System of MOF and MARA (CARS-46) and the Central Public-interest Scientific Institution Basal Research Fund, CAFS (No. 2020TD32). The funding agency played no part in the study design, data collection and analysis, decision to publish, or manuscript preparation.

## Conflict of Interest

CL was employed by the company Xinjiang Tianyun Organic Agriculture Co., Yili Group. The remaining authors declare that the research was conducted in the absence of any commercial or financial relationships that could be construed as a potential conflict of interest.

## Publisher's Note

All claims expressed in this article are solely those of the authors and do not necessarily represent those of their affiliated organizations, or those of the publisher, the editors and the reviewers. Any product that may be evaluated in this article, or claim that may be made by its manufacturer, is not guaranteed or endorsed by the publisher.

## Supplementary Material

Supplementary Figure S1 The correlation of samples for next generation sequencing.

Supplementary Figure S2 The standard curves of Elisa kits for GnRH3, LH, FSH, and E2 in lenok.

Supplementary Table S1 The analysis of SSR by MISA.

Supplementary Table S2 The information about the DEGs enriched in pathways involved in endocrine systems and developments.

Supplementary Table S3 The primer information of DEGs selected for qRT-PCR verification.

Supplementary Table S4 The results of intra-assay repeat tests of ELISA.
