# Supplementary figures and images for: Screening and Validation of p38 MAPK Involved in Ovarian Development of Brachymystax lenok
Source: Front Vet Sci. 2022 Feb 16;9:752521. doi: 10.3389/fvets.2022.752521 (PMC8889577; doi:10.3389/fvets.2022.752521)

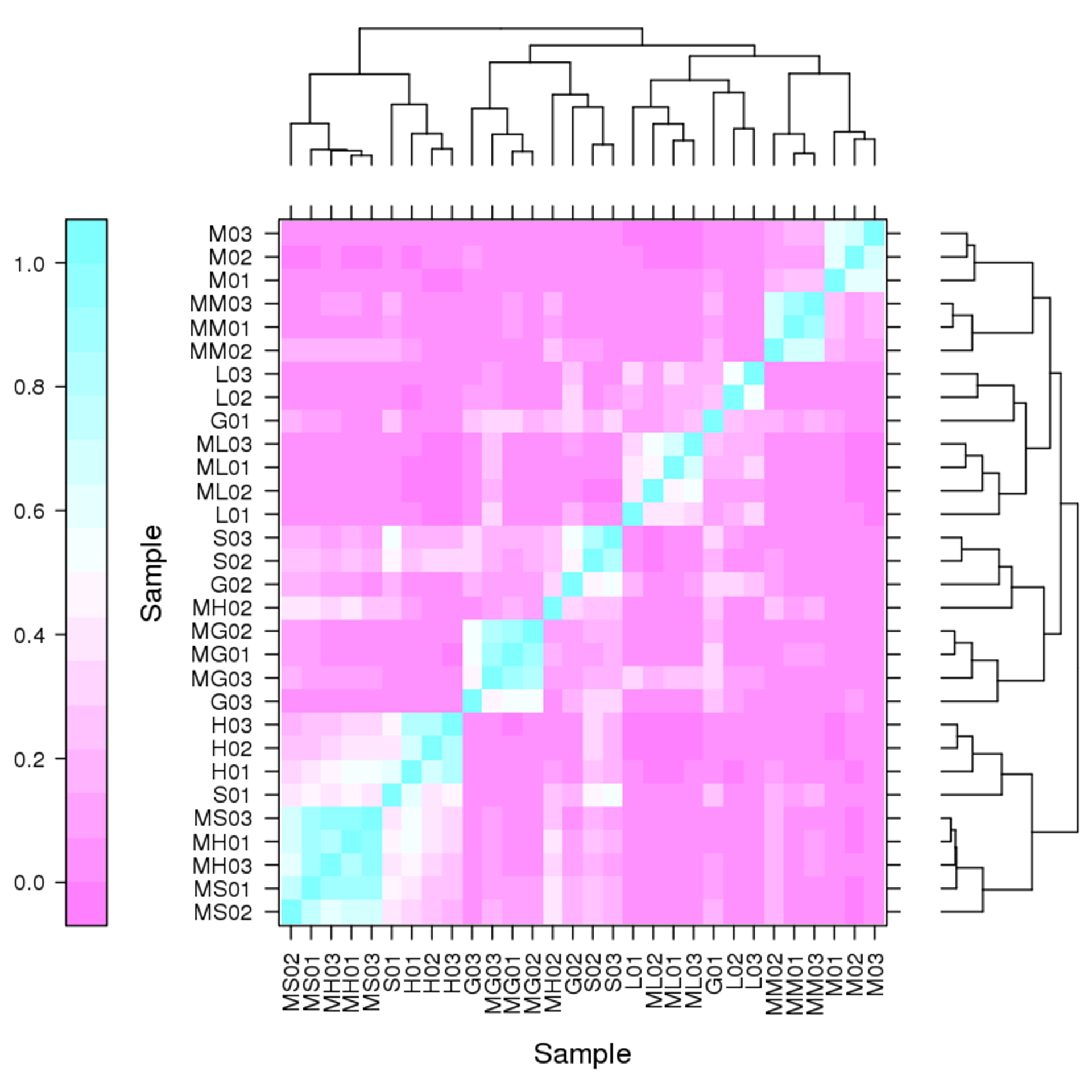

Supplement: Supplementary Figure S1 — The correlation of samples for next generation sequencing. [file Image_1.PNG]

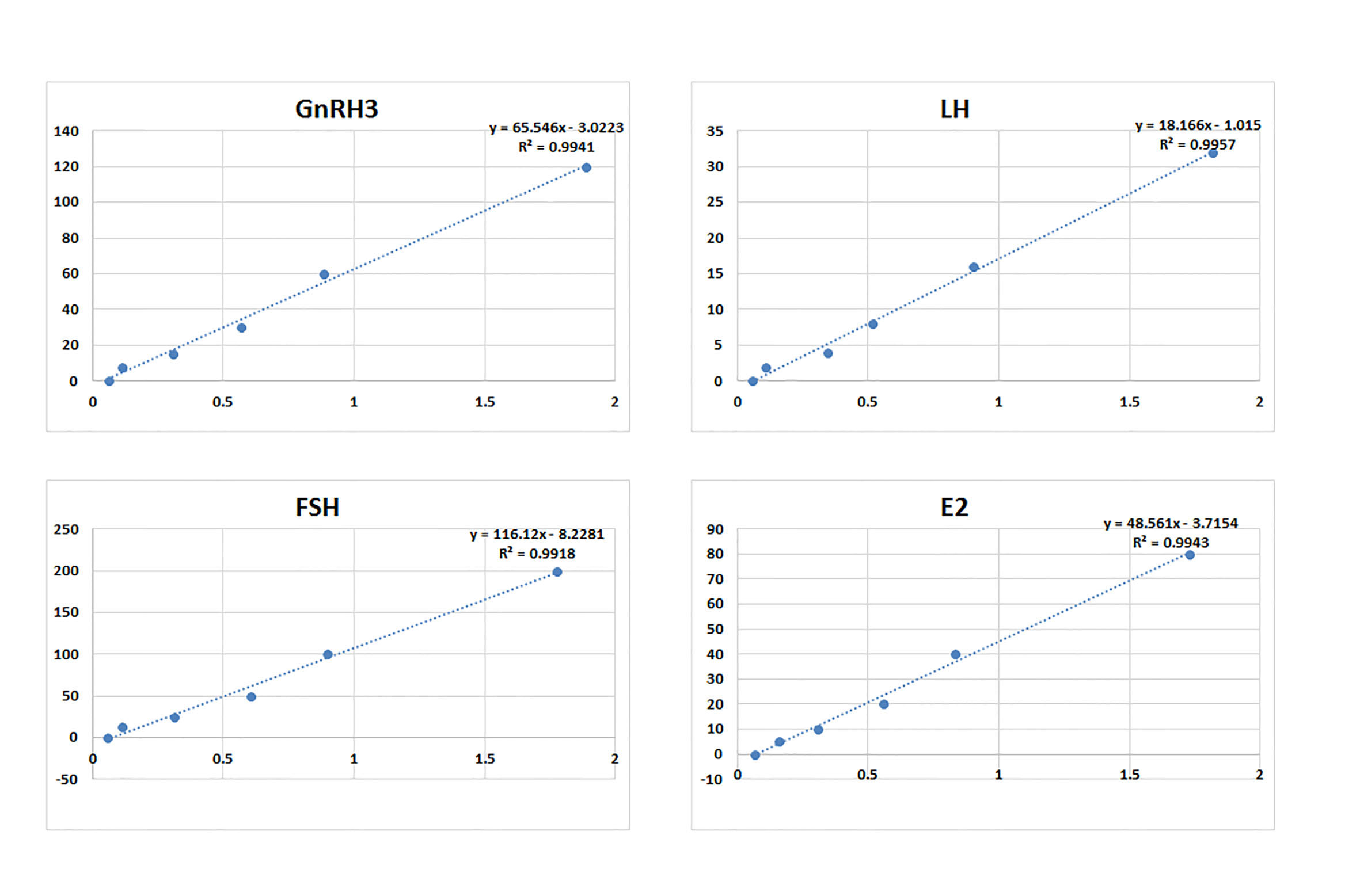

Supplement: Supplementary Figure S2 — The standard curves of Elisa kits for GnRH3, LH, FSH, and E2 in lenok. [file Image_2.JPEG]
