# Supplementary material for: Screening and Validation of p38 MAPK Involved in Ovarian Development of Brachymystax lenok
Source: Front Vet Sci. 2022 Feb 16;9:752521. doi: 10.3389/fvets.2022.752521 (PMC8889577; doi:10.3389/fvets.2022.752521)
Supplement: Supplementary Table S1 — The analysis of SSR by MISA. [file Table_1.DOCX]

Table S1 The analysis of SSR by MISA

| Searching item | Numbers |
| --- | --- |
| Total number of sequences examined | 104,373 |
| Total size of examined sequences (bp) | 344,196,344 |
| Total number of identified SSRs | 103,688 |
| Number of SSR containing sequences | 49,478 |
| Number of sequences containing more than 1 SSR | 23,300 |
| Number of SSRs present in compound formation | 21,101 |
| Mono nucleotide | 42,411 |
| Di nucleotide | 46,227 |
| Tri nucleotide | 12,599 |
| Tetra nucleotide | 2,169 |
| Penta nucleotide | 162 |
| Hexa nucleotide | 120 |
