# Supplementary material for: Screening and Validation of p38 MAPK Involved in Ovarian Development of Brachymystax lenok
Source: Front Vet Sci. 2022 Feb 16;9:752521. doi: 10.3389/fvets.2022.752521 (PMC8889577; doi:10.3389/fvets.2022.752521)
Supplement: Supplementary Table S3 — The primer information of DEGs selected for qRT-PCR verification. [file Table_3.DOCX]

Table S3 The primer information of DEGs selected for qRT-PCR verification

| Gene ID | | primer sequence (5’-3’) |
| --- | --- | --- |
| F01_transcript_55235 | F | GTTAGGTTTTCCCGTGGCAG |
|  | R | GATCAACCTGATAATGTT |
| F01_transcript_10204 | F | CTGCCCCAACTCCACCAAC |
|  | R | AAGCTGCGAAGCATCAACTGT |
| F01_transcript_29849 | F | CCTGGACGAGAGGAAGGAGA |
|  | R | CCGTGGTAGATGTAGACGGC |
| F01_transcript_33296 | F | TCCTCCGAGACCTCTACACA |
|  | R | CTCCAGCGTCTTCTCAATGC |
| F02_transcript_5540 | F | CTGAACTCCTCACTGGCAGA |
|  | R | CCTTGCAGACACTGAGGAGA |
| F01_transcript_71088 | F | CCTCTCAGCTACCCACAGAC |
|  | R | AAGGGTGTACGAGGACAGTG |
| F01_transcript_1489 | F | TCATGCAGCACAGGAGGTAA |
|  | R | ATATTGGGGCGGATGGGTAG |
| F02_transcript_76442 | F | TTGTGTTTAATCGGCGAGTC |
|  | R | CTGACAGTTCCCAGTCCAGA |
| F02_transcript_21613 | F | AACCCTCCATCTTGTGCTCC |
|  | R | TCTTGGCCTTGACATTCTCG |
| F02_transcript_6599 | F | CATCGAGAAGGGCAAGAAGC |
|  | R | GAAACACAGCAGGACCACAG |
| F01_transcript_41574 | F | CATTGACATCATGAGGGCCG |
|  | R | ATGGGGAACAGGGAAGAGTG |
| F01_transcript_44977 | F | TCCTGTTGGAATTGGAGGCT |
|  | R | AGGAACAGGTAGTTGGCCTC |
