# Supplementary material for: Screening and Validation of p38 MAPK Involved in Ovarian Development of Brachymystax lenok
Source: Front Vet Sci. 2022 Feb 16;9:752521. doi: 10.3389/fvets.2022.752521 (PMC8889577; doi:10.3389/fvets.2022.752521)
Supplement: Supplementary Table S4 — The results of intra-assay repeat tests of ELISA. [file Table_4.DOCX]

Table S4 The results of intra-assay repeat tests of ELISA

|  | | Serum sample and value of OD450 | | | | | |
| --- | --- | --- | --- | --- | --- | --- | --- |
|  |  | Times of repetition | | | | | |
|  |  | 1 | 2 | 3 | Mean value | Standard deviations | variable coefficient |
| GnRH  CK | Sample 1 | 0.426 | 0.465 | 0.414 | 0.435 | 0.026665 | 0.061298 |
|  | Sample 2 | 0.377 | 0.385 | 0.362 | 0.374667 | 0.011676 | 0.031164 |
|  | Sample 3 | 0.414 | 0.394 | 0.427 | 0.411667 | 0.016623 | 0.04038 |
| GnRH  DMSO | Sample 1 | 0.145 | 0.156 | 0.149 | 0.15 | 0.00455 | 0.03033 |
|  | Sample 2 | 0.285 | 0.296 | 0.254 | 0.278333 | 0.021779 | 0.078249 |
|  | Sample 3 | 0.198 | 0.187 | 0.209 | 0.198 | 0.011 | 0.055556 |
| GnRH  DMSO+I | Sample 1 | 0.614 | 0.596 | 0.559 | 0.589667 | 0.028042 | 0.047555 |
|  | Sample 2 | 0.598 | 0.568 | 0.571 | 0.579 | 0.016523 | 0.028537 |
|  | Sample 3 | 0.615 | 0.636 | 0.624 | 0.625 | 0.010536 | 0.016857 |
| FSH  CK | Sample 1 | 0.596 | 0.574 | 0.582 | 0.584 | 0.011136 | 0.019068 |
|  | Sample 2 | 0.496 | 0.473 | 0.465 | 0.478 | 0.016093 | 0.033668 |
|  | Sample 3 | 0.562 | 0.548 | 0.539 | 0.549667 | 0.01159 | 0.021086 |
| FSH  DMSO | Sample 1 | 0.325 | 0.320 | 0.291 | 0.312 | 0.018358 | 0.058838 |
|  | Sample 2 | 0.366 | 0.347 | 0.383 | 0.365333 | 0.018009 | 0.049295 |
|  | Sample 3 | 0.337 | 0.340 | 0.398 | 0.358333 | 0.034385 | 0.095958 |
| FSH  DMSO+I | Sample 1 | 0.256 | 0.229 | 0.243 | 0.242667 | 0.013503 | 0.055645 |
|  | Sample 2 | 0.198 | 0.182 | 0.169 | 0.183 | 0.014526 | 0.079376 |
|  | Sample 3 | 0.182 | 0.195 | 0.175 | 0.184 | 0.010149 | 0.055157 |
| LH  CK | Sample 1 | 0.758 | 0.764 | 0.795 | 0.772333 | 0.019858 | 0.025711 |
|  | Sample 2 | 0.715 | 0.726 | 0.707 | 0.716 | 0.009539 | 0.013323 |
|  | Sample 3 | 0.659 | 0.664 | 0.682 | 0.668333 | 0.012097 | 0.0181 |
| LH  DMSO | Sample 1 | 0.541 | 0.532 | 0.578 | 0.550333 | 0.024379 | 0.044299 |
|  | Sample 2 | 0.525 | 0.514 | 0.492 | 0.510333 | 0.016803 | 0.032925 |
|  | Sample 3 | 0.496 | 0.475 | 0.473 | 0.481333 | 0.012741 | 0.02647 |
| LH  DMSO+I | Sample 1 | 0.359 | 0.328 | 0.337 | 0.341333 | 0.015948 | 0.046722 |
|  | Sample 2 | 0.291 | 0.302 | 0.308 | 0.300333 | 0.008622 | 0.028707 |
|  | Sample 3 | 0.303 | 0.290 | 0.283 | 0.292 | 0.010149 | 0.034756 |
| E2  CK | Sample 1 | 0.789 | 0.758 | 0.767 | 0.771333 | 0.015948 | 0.020676 |
|  | Sample 2 | 0.821 | 0.768 | 0.817 | 0.802 | 0.029513 | 0.036799 |
|  | Sample 3 | 0.767 | 0.785 | 0.794 | 0.782 | 0.013748 | 0.01758 |
| E2  DMSO | Sample 1 | 0.578 | 0.599 | 0.580 | 0.585667 | 0.01159 | 0.01979 |
|  | Sample 2 | 0.567 | 0.547 | 0.570 | 0.561333 | 0.012503 | 0.022274 |
|  | Sample 3 | 0.628 | 0.659 | 0.630 | 0.639 | 0.017349 | 0.027151 |
| E2  DMSO+I | Sample 1 | 0.387 | 0.449 | 0.438 | 0.424667 | 0.033081 | 0.077898 |
|  | Sample 2 | 0.298 | 0.287 | 0.283 | 0.289333 | 0.007767 | 0.026846 |
|  | Sample 3 | 0.212 | 0.237 | 0.235 | 0.228 | 0.013892 | 0.060932 |
